# New Perspectives on Lactose Malabsorption, Celiac Disease and Related Disorders

**DOI:** 10.3390/nu15112512

**Published:** 2023-05-29

**Authors:** Paolo Usai-Satta, Mariantonia Lai

**Affiliations:** 1 Gastroenterology Unit, ARNAS G. Brotzu, Piazza Ricchi 1, 09131 Cagliari, Italy; 2 Digestive Endoscopy, ARNAS G. Brotzu, Piazza Ricchi 1, 09131 Cagliari, Italy; laimariantonia@gmail.com

Lactose malabsorption (LM) is caused by the incomplete hydrolysis of lactose due to lactase deficiency. LM is a well-known cause of abdominal symptoms classified as lactose intolerance. In addition, lactose is a fundamental component of fermentable oligo-, di-, and monosaccharides and polyols (FODMAPs), and is consequently related to intolerance to these compounds [1,2].

Celiac disease (CD) is a permanent, chronic, gluten-sensitive disorder characterized by small intestinal inflammation and malabsorption in genetically predisposed individuals. LM and CD can be mutually associated. A transient lactase deficiency is present in CD on a normal diet. The persistence of symptoms in CD on a gluten-free diet (GFD) may be instead, in part, attributed to primary LM. Similar circumstances are present in inflammatory bowel diseases (IBDs), in which LM can be responsible for some persistent symptoms of IBDs on clinical remission. LM and irritable bowel syndrome (IBS) are instead independent conditions where a lactose-restricted diet may be useful for some IBS patients [1].

This Special Issue, entitled “Nutrition in patients with lactose malabsorption, celiac disease and related disorders”, comprises seven peer-reviewed papers reporting on several clinical and nutritional aspects related to these clinical conditions.

In her paper, Alkalay [2] provides an overview of the nutritional problems related to LM, CD, gluten-related disorders, and FODMAP intolerance. Although LM does not cause a direct calcium malabsorption, a lactose-restricted diet with a dietary avoidance of dairy products can lead to sub-optimal bone mineralization.

Common nutritional deficiencies in untreated CD are iron, B12, calcium, Vitamin D, and zinc. In most CD patients, a strict GFD should result in complete resolution of their symptoms. However, despite a GFD, nutritional deficiencies can persist in approximately 30% of CD patients.

Regarding a low-FODMAP diet, the risk of depletion in fiber, calcium, protein, iron, and Vitamin B12 is well known, and the role of a trained dietician should be considered fundamental.

Similarly, the aim of the study by Jasielska et al. [3] was to determine the incidence of hypocalcemia and vitamin D deficiency in children with IBDs and associated LM. LM was diagnosed in 23.2% children with Crohn’s disease and in 22.6% with ulcerative colitis. There was no difference in the concentrations of total calcium, phosphorus, and vitamin D between patients on a low-lactose diet and normal diet. In conclusion, a lactose-restricted diet had no effect on bone mineral density in patients with IBDs and associated LM. 

Additionally, Farina et al. [4] analyzed the clinical significance of anti-tissue transglutaminase antibodies (tTGA) in a cohort of treated CD patients with persistently positive serology. Overall, the tTGA titres were three-fold increased. Three different tTGA trends were recognized: 55% of patients with a progressive titre decrease; 25% with a fluctuating behavior; and 20% patients with a steady state or increased titres. However, tTGA positivity during CD follow-up did not demonstrate clinical significance without association with autoimmune comorbidities and mucosal damage.

The article by De Angelis et al. [5] aimed to define the microbial consortia that are able to digest gluten into non-toxic and non-immunogenic peptides in the human gastrointestinal tract. A total of 190 strains of the genus *Bacillus* and 314 lactic acid bacteria were evaluated. Consortium activity was evaluated by ELISA tests and duodenal explants from CD patients. In this experimental model, two specific microbial consortia were able to hydrolyze gluten and could potentially improve the digestion of gluten in gluten-sensitive patients.

The study by Rollet et al. [6] investigated the association between dietary patterns and bowel movements in adults living in Luxembourg. Data from 1431 participants who completed a 174-item food-frequency questionnaire were analyzed. Interestingly, neither fruits and vegetables were significantly associated with constipation. In particular grains, lipid-rich foods, total fats, and starch were associated with a lower constipation score, while sugary products were associated with higher constipation.

Although constipation is not usually associated with LM, the paper by Leszkowicz et al. [7] provides a narrative review on LM, its epidemiology and pathogenesis, and the potential correlation between lactose intolerance and constipation in children. Constipation could be particularly prevalent in non-hydrogen-producing children with LM and may be related to methane produced by gut bacteria.

In conclusion, the different perspectives presented in this Special Issue confirm that the clinical and nutritional aspects of LM, CD, and related disorders are a current challenging scientific topic. We would like to thank all the authors and the editorial team of *Nutrients* for their contributions.
